# A computed tomography urography‐based machine learning model for predicting preoperative pathological grade of upper urinary tract urothelial carcinoma

**DOI:** 10.1002/cam4.6901

**Published:** 2024-01-04

**Authors:** Yanghuang Zheng, Hongjin Shi, Shi Fu, Haifeng Wang, Jincheng Wang, Xin Li, Zhi Li, Bing Hai, Jinsong Zhang

**Affiliations:** ^1^ Department of Urology The 2nd Affiliated Hospital of Kunming Medical University Kunming Yunnan People's Republic of China; ^2^ Department of Urology The First People's Hospital of Luliang County Lijiang Yunnan People's Republic of China; ^3^ Department of Urology The Cancer Hospital of Yunnan Province Kunming Yunnan People's Republic of China; ^4^ Department of Radiology The First People's Hospital of Yunnan Province Kunming Yunnan People's Republic of China; ^5^ Department of Respiratory Medicine The 2nd Affiliated Hospital of Kunming Medical University Kunming Yunnan People's Republic of China

**Keywords:** carcinoma, machine learning, pathological grade, radiomics, urinary tract

## Abstract

**Objectives:**

Development and validation of a computed tomography urography (CTU)‐based machine learning (ML) model for prediction of preoperative pathology grade of upper urinary tract urothelial carcinoma (UTUC).

**Methods:**

A total of 140 patients with UTUC who underwent CTU examination from January 2017 to August 2023 were retrospectively enrolled. Tumor lesions on the unenhanced, medullary, and excretory periods of CTU were used to extract Features, respectively. Feature selection was screened by the Pearson and Spearman correlation analysis, least absolute shrinkage and selection operator algorithm, random forest (RF), support vector machine (SVM), and eXtreme Gradient Boosting (XGBoost). The logistic regression (LR) was used to screen for independent influencing factors of clinical baseline characteristics. Machine learning models based on different feature datasets were constructed and validated using algorithms such as LR, RF, SVM, and XGBoost. By computing the selected features, a radiomics score was generated, and a diverse feature dataset was constructed. Based on the training set, 16 ML models were created, and their performance was evaluated using the validation set for metrics including sensitivity, specificity, accuracy, area under the receiver operating characteristic curve (AUC), and others.

**Results:**

The training set consisted of 98 patients (mean age: 64.5 ± 10.5 years; 30 males), whereas the validation set consisted of 42 patients (mean age: 65.3 ± 9.78 years; 17 males). Hydronephrosis was the best independent influence factor (*p* < 0.05). The RF model had the best performance in predicting high‐grade UTUC, with AUC of 0.914 (95% Confidence Interval [95%CI] 0.852–0.977) and 0.903 (95%CI 0.809–0.997) in the training set and validation set, and accuracy of 0.878 and 0.857, respectively.

**Conclusions:**

An ML model based on the RF algorithm exhibits excellent predictive performance, offering a non‐invasive approach for predicting preoperative high‐grade UTUC.

## Abstract 2

The radiomics‐based machine learning model developed in our research. It has good performance in predicting the pathological grade of UTUC and is expected to become an important supplementary tool for traditional tissue biopsy.

## 1 INTRODUCTION

Upper urinary tract urothelial carcinoma (UTUC) is a relatively uncommon malignant neoplasm that arises in the renal pelvis and ureter, constituting merely 5%–10% of all cases of urothelial carcinoma.^1^ UTUC is predominantly observed in individuals aged between 70 and 90 years, exhibiting a twofold higher prevalence among males.^2^ Established prognostic factors such as tumor stage and pathological grade play significant roles in predicting outcomes for UTUC.^3^, ^4^ Different tumor grades can show varying degrees of aggressiveness. Previous study has shown that pathological grade is an independent influencing factor of specific mortality in patients with UTUC.^5^ There has a study also demonstrated a significant positive correlation (*p* < 0.01) between postoperative pathological grade and 5‐year mortality in UTUC patients.^6^ Hence, precise preoperative identification of the tumor's pathological grade is essential to guide subsequent treatment strategies effectively.^7^

Current diagnostic methods for UTUC encompass computed tomography urography (CTU), magnetic resonance imaging (MRI), urine cytology, and other techniques. The preferred radiological examination for diagnosing UTUC is CTU. A meta‐analysis demonstrated that the sensitivity and specificity of CTU in UTUC diagnosis were 92% and 95%, respectively.^8^ Nevertheless, the determination of tumor pathological grade based on CTU examination remains challenging. Although ureteroscopy combined with ureteroscopic biopsy can assist in the clarification of disease diagnosis, challenges persist in specimen collection and difficulties arise from biopsy technology, impeding the accuracy of pathological grade and tumor stage.^9^ Additionally, it is crucial to note that ureteroscopy or biopsy presents a risk factor for intravesical recurrence of UTUC.^10^

The aggressiveness of tumors is often linked to heterogeneity, a vital characteristic of malignant tumors.^11^, ^12^ Radiomics technology enables the assessment of tumor image heterogeneity acquired during routine clinical practice.^13^, ^14^ By extracting and evaluating features from digital images, it facilitates the detection of subtle changes and heterogeneity that may be imperceptible to the naked eye, thereby offering a novel approach for tumor pathological grade diagnosis.^15^ The utilization of CT images for the extraction of radiomics features has been demonstrated to differentiate the pathological grade of renal cell carcinoma effectively.^16^ Despite its widespread application in various tumor diseases, its utilization remains limited in UTUC.

The objective of this study is to extract radiomics features from CTU images and develop a non‐invasive radiomics‐based machine learning (ML) model capable of predicting the pathology of high‐grade UTUC. This model aims to assist in clinical disease diagnosis, provide clarity on pathological grade, and offer potential alternatives to ureteroscopy and biopsy techniques.

## 2 MATERIALS AND METHODS

### 2.1 Patients

The patients diagnosed with UTUC from the Second Affiliated Hospital of Kunming Medical University between January 2017 and August 2023 were retrospectively included as both the training and validation sets for developing ML model. Inclusion criteria included the following: (1) Patients diagnosed with UTUC through pathological examination following radical nephroureterectomy; (2) CTU images conducted within 1 month before the surgical procedure; (3) A minimum of three urine cytology examinations performed within 2 weeks preceding the surgery. Exclusion criteria included the following: (1) Coexistence with other tumors; (2) Preoperative neoadjuvant therapy; (3) Difficulty in obtaining CTU images; (4) Difficulty in segmenting the tumor lesions; (5) No CTU examination was performed before surgery. This retrospective study was approved by the Institutional Review Board of the Second Affiliated Hospital of Kunming Medical University, and informed consent was waived.

**FIGURE 1:**
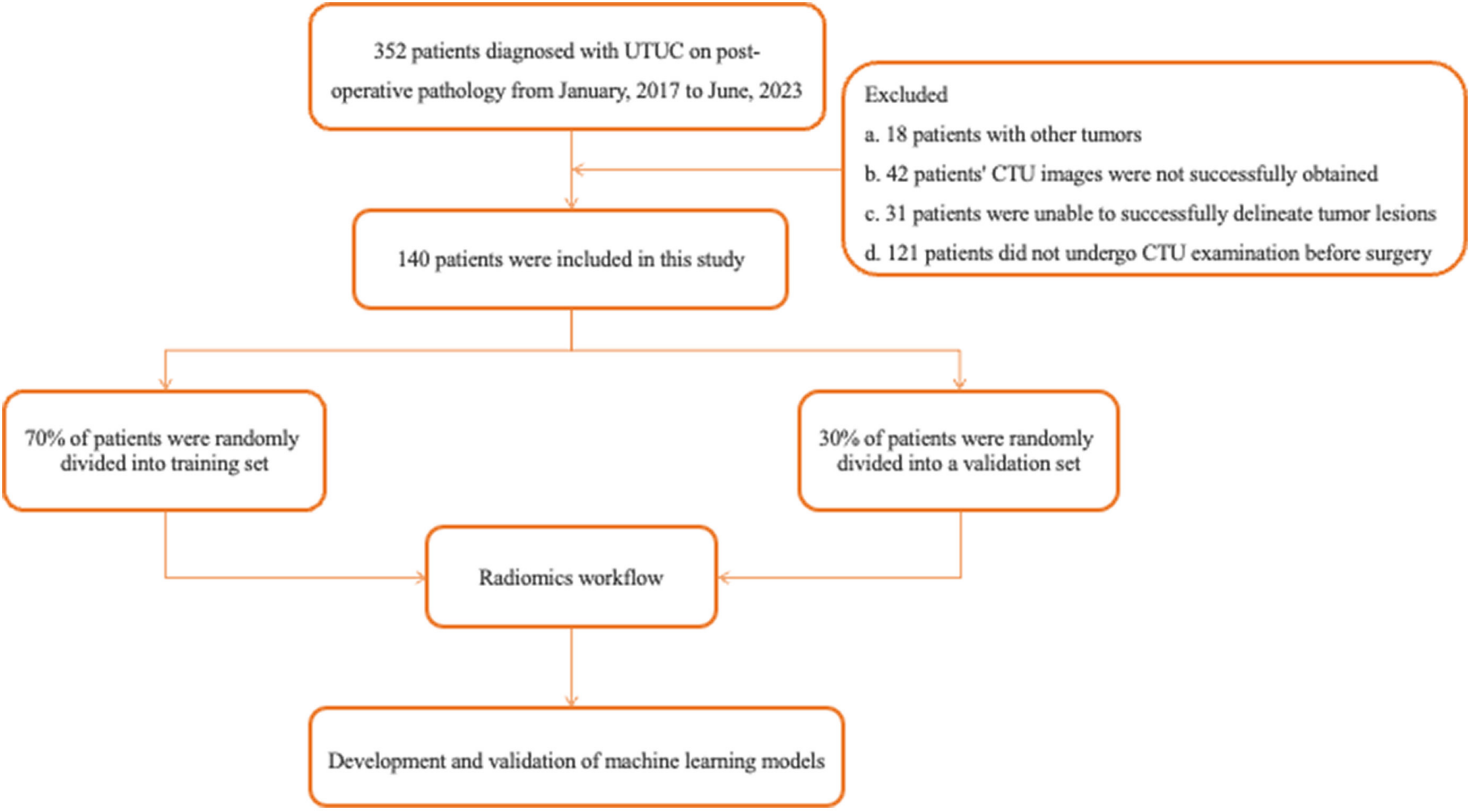
Flowchart of the study.

A total of 140 patients who met the inclusion and exclusion criteria were included in both the training set and validation set (Figure 1). The clinical baseline characteristics collected included the patient's T‐stage, age, gender, hydronephrosis, urine cytology, tumor location, and tumor type.

### 2.2 Radiomics workflow

**FIGURE 2:**
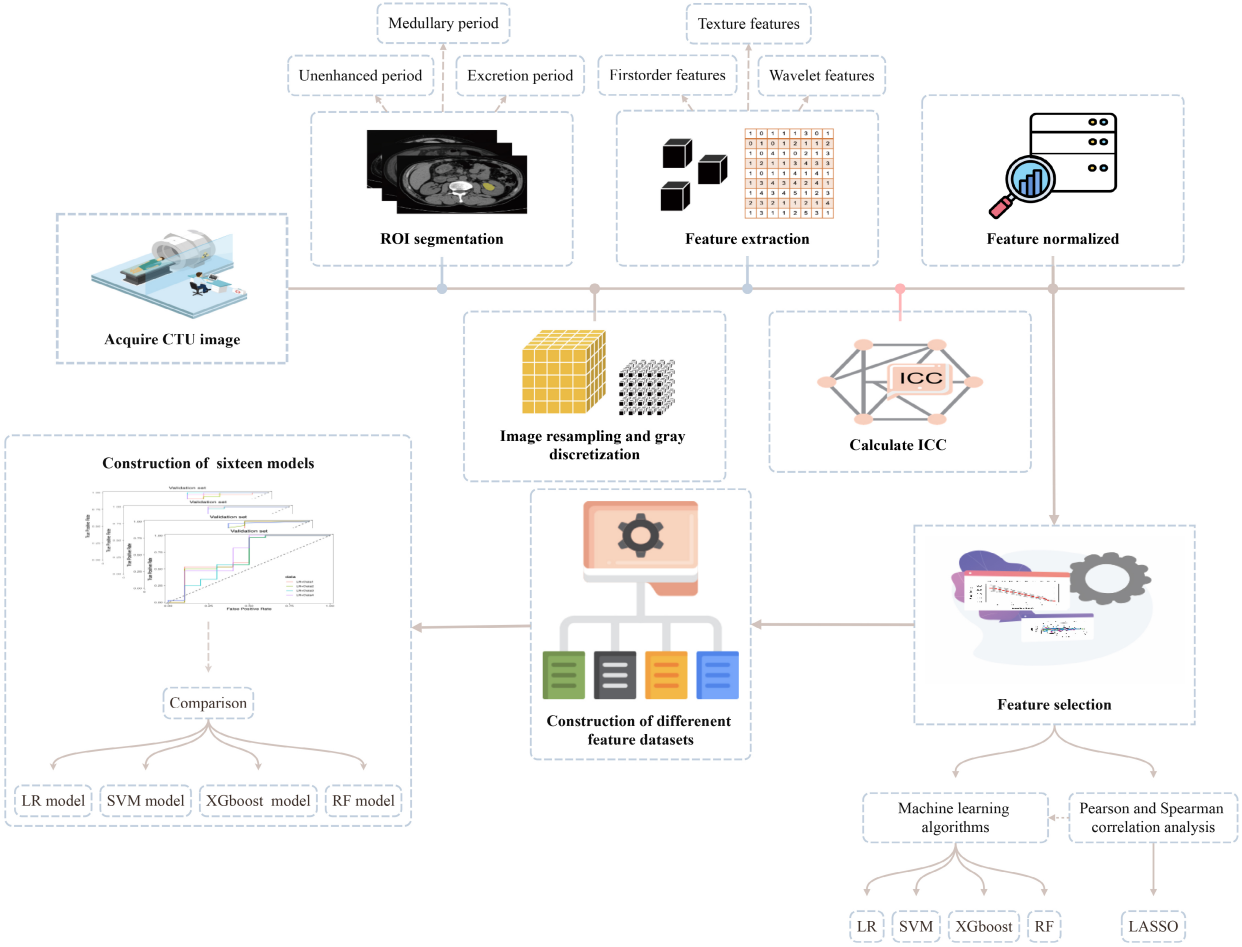
Radiomics workflow.

The CTU image acquisition process is described in detail in the Appendix S1, while the radiomics workflow is illustrated in Figure 2.

### 2.3 Urine cytological examination and pathological grading

All patients underwent a minimum of three urine cytology examinations within 2 weeks before surgery. The procedure and results of cytology examinations are described in the Appendix S1. Urinary cytologic samples from UTUC patients in our study population were independently reexamined by two pathologists. The result of urine cytology was based on the Paris System classified as either negative (Paris 1–3,6) or positive (Paris 4,5). Pathological grade was based on the WHO 2004/2016 classification system, which included papillary urothelial neoplasms of low malignant potential (PUNLMP), low‐grade urothelial carcinoma, and high‐grade urothelial carcinoma. Inconsistent reports were resolved by a third senior pathologist. Pathological grades were classified as either low‐grade (PUNLMP and low‐grade urothelial carcinoma) or high‐grade (high‐grade urothelial carcinoma).

### 2.4 Image segmentation

The 3D slicer software (version 5.0.3; www.slicer.org) was used to segment the two‐dimensional region of interest (2D‐ROI) with maximum cross‐sectional area in the unenhanced period, medullary period, and excretion period of CTU image respectively. For each segmentation lesion, a senior radiologist with 10 years of experience in urogenital imaging guided manual segmentation to ensure accurate tumor margins. 2D‐ROI is located at a distance of 1 mm from the tumor margin. Segmentation of tumors on unenhanced period images was performed based on the excretion period images.

### 2.5 Extraction and selection of features

Feature extraction from 2D‐ROI was performed using the Radiomics module (version 3.0.1) of the 3D Slicer software. CTU image was resampled into isotropic voxels of 3 × 3 × 3 mm^3^ using spline interpolation. Hounsfield units (HU) were discretized to a specific value of 25 HU. After resampling and gray discretization, the image underwent feature extraction. Extracted features encompass firstorder features (18 features), texture features including gray‐level co‐occurrence matrix (GLCM) (24 features), gray‐level dependence matrix (GLDM) (14 features), gray‐level runlength matrix (GLRLM) (16 features), gray‐level size‐zone matrix (GLSZM) (16 features), neighboring gray‐tone difference matrix (NGDTM) (5 features), and wavelet features (consisting of L‐H‐H, H‐H‐H, L‐L‐H, H‐L‐H, L‐H‐L, H‐H‐L, L‐L‐L, and H‐L‐L).

To assess the intraobserver reproducibility, 30 patients were randomly selected for segment ROI segmentation again after 1 month. Intraclass correlation coefficient (ICC) was used to evaluate the intraobserver consistency of feature extraction, in which ICC > 0.75 indicates good, and relevant features with ICC < 0.75 were excluded.^17^, ^18^ The remaining features were normalized using Z‐score, which involved subtracting their mean and dividing by their standard deviation (SD). The normalized features were subjected to a normality test, followed by Pearson correlation analysis for the normally distributed features and Spearman correlation analysis for the non‐normally distributed features. The least absolute shrinkage and selection operator algorithm (LASSO) algorithm was used to select the remaining features subsequently. What's more, ML algorithms of support vector machine (SVM), random forest (RF), and eXtreme Gradient Boosting (XGboost) were employed to select features after normalization again to ensure the selection of optimal features. The univariate logistic regression (LR) and multivariate LR algorithm to identify the best independent influence factor among the clinical baseline characteristics. The radiomics score was calculated by a linear combination of features weighted by the noose coefficient. The formula was as follows:

$$\text{Radiomics score}=\sum_{i=1}^{n}\mathrm{Coe}f_{i}\times x_{i}$$

where, *x*_i_ is the normalized value of each selected feature and Coe*f*_i_ is the coefficient corresponding to each selected feature.^19^

### 2.6 Construction of different feature dataset

The construction of four feature datasets was performed. First, the general‐feature dataset was generated by selecting features with ICC > 0.75 and further screened using Pearson and Spearman correlation analysis and LASSO algorithm. Second, the clinical‐feature dataset was established by adding the best independent influence factor (*p* < 0.05) to the general‐feature dataset. Third, the clinical‐radiomics feature dataset was established by combining the best independent influencing factors with the radiomics score calculated by the general‐feature dataset. Last, the optimal clinical‐radiomics feature dataset was constructed by integrating key features identified through SVM, RF, and XGboost algorithms with the clinical‐radiomics feature dataset.

### 2.7 Constructing diverse ML models

Four kinds of feature datasets were utilized to construct ML models employing LR, SVM, RF, and XGBoost algorithms. The SVM, RF, and XGBoost ML models underwent optimization through grid search. Subsequently, the training set and validation set were evaluated for the area under the receiver operating characteristic curve (AUC), sensitivity, specificity, accuracy, and 95% Confidence Interval (95% CI).

### 2.8 Statistical analysis

All statistical analyses were performed with R Studio (version 4.2.2) and Python software (version 3.7.3). Data conforming to a normal distribution were presented as mean ± standard deviation (x ± s), and group comparisons were analyzed using the independent‐sample *t*‐test. Non‐normally distributed data were expressed as median (upper and lower quartiles), and group comparisons were assessed using the Mann–Whitney *U*‐test. Categorical variables were presented as constituent ratios or rates (%) and compared between groups using the chi‐squared test (χ^2^) or Fisher's exact test. Statistical significance was defined as *p* < 0.05.

## 3 RESULTS

### 3.1 Patient characteristics

In this study, a total of 140 patients with UTUC were included and randomly assigned to the training set (*n* = 98) and the validation set (*n* = 42) in a 7:3 ratio. Among them, there were 47 male patients and 93 female patients, with an average age of 64.7 ± 10.28 years. It consisted of 33 patients (23.6%) with low‐grade pathology and 107 patients (76.4%) with high‐grade pathology. In the training set, there were 23 patients (23.5%) with low‐grade pathology and 75 patients (76.5%) with high‐grade pathology; whereas in the validation set, 10 patients (23.8%) with low‐grade pathology and 32 patients (76.2%) with high‐grade pathology respectively. T‐stage, hydronephrosis and urine cytology showed statistically significant differences between low‐grade UTUC and high‐grade UTUC in both the training set and the validation set respectively (*p* < 0.05). However, no statistically significant differences were observed in other clinical baseline characteristics among these two sets (all *p* > 0.05) (Table 1).

**TABLE 1:** Patient clinical characteristics.

Characteristics	Training set (*n* = 98)			Validation set (*n* = 42)		
	Low grade	High grade	*p*‐value	Low grade	High grade	*p*‐value
T‐stage			<0.001			0.007
Ta, no. (%)	8 (34.8%)	7 (9.3%)		4 (40.0%)	6 (18.8%)	
Tis, no. (%)	2 (8.7%)	1 (1.3%)		1 (10.0%)	0 (0.0%)	
T1, no. (%)	8 (34.8%)	11(14.7%)		4 (40.0%)	4 (12.5%)	
T2, no. (%)	2 (8.7%)	24 (32.0%)		0 (0.0%)	10 (31.2%)	
T3, no. (%)	3 (13.0%)	32 (42.7%)		1 (10.0%)	12 (37.5%)	
Hydronephrosis			0.015			0.032
No, no. (%)	7 (30.4%)	6 (8.0%)		4 (40.0%)	2 (6.2%)	
Yes, no. (%)	16 (69.6%)	69 (92.0%)		6 (60.0%)	30 (93.8%)	
Urine cytology			0.033			0.010
Negative, no. (%)	16 (69.6%)	31 (41.3%)		8 (80.0%)	10 (31.2%)	
Positive, no. (%)	7 (30.4%)	44 (58.7%)		2 (20.0%)	22 (68.8%)	
Gender			0.983			0.686
Female (no. (%))	16 (69.6%)	52 (69.3%)		7 (70.0%)	18 (56.2%)	
Male (no. (%))	7 (30.4%)	23 (30.7%)		3 (30.0%)	14 (43.8%)	
Age (mean ± SD)	64.0 ± 9.23	64.7 ± 10.9	0.771	66.6 ± 10.5	64.8 ± 9.68	0.645
Tumor location			0.315			0.565
Left (no. (%))	15 (65.2%)	40 (53.3%)		7 (70.0%)	17 (53.1%)	
Right, no. (%)	8 (34.8%)	35 (46.7%)		3 (30.0%)	15 (46.9%)	
Tumor type			0.455			0.875
Renal pelvic, no. (%)	14 (60.9%)	39 (52.0%)		5 (50.0%)	13 (40.6%)	
Ureter, no. (%)	9 (39.1%)	36 (48.0%)		5 (50.0%)	19 (59.4%)	

*Note*: *p*‐value, χ^2^ or Fisher's exact test.

### 3.2 Feature selection and construction of feature datasets

**FIGURE 3:**
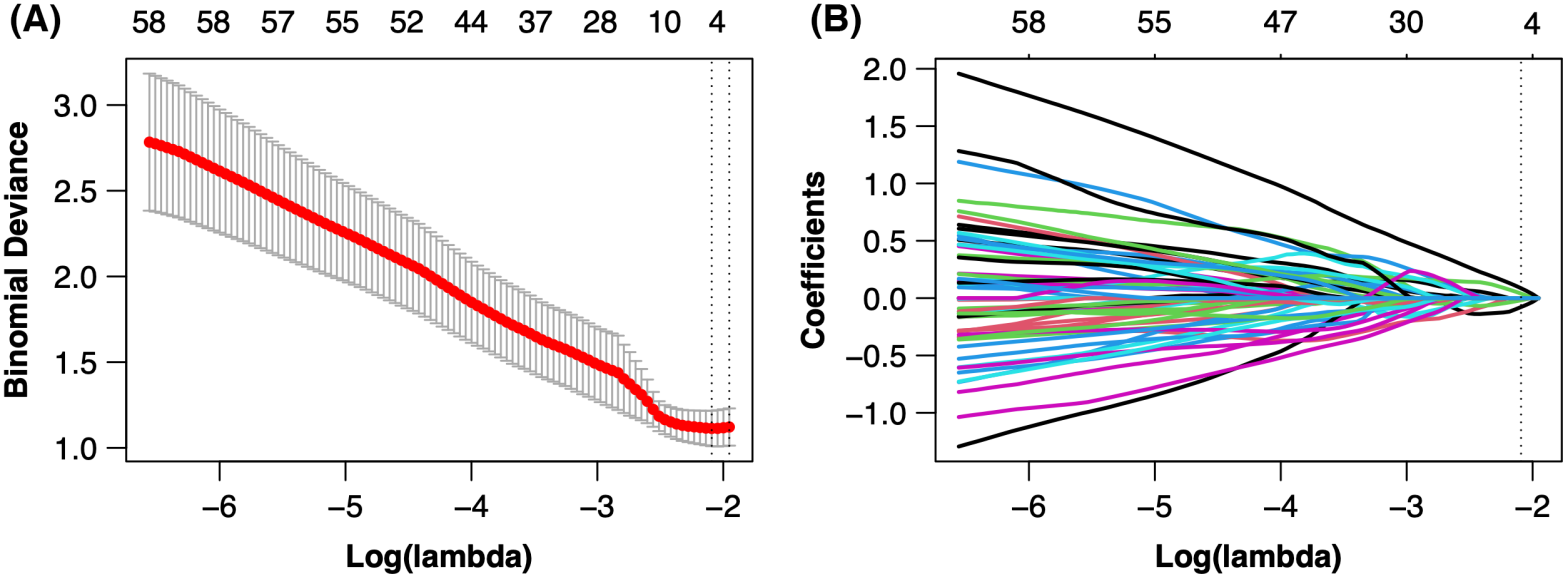
Feature selection using the least absolute shrinkage and selection operator algorithm (LASSO) algorithm. (A) Figure of binomial deviation versus logarithm (λ). 5‐fold cross validation to confirm the optimal tuning parameter (*λ*), *λ*_min_ = 0.123, log (λ) = −2.096. (B) Figure of the lasso coefficients versus logarithm (*λ*). Four features with the lowest lasso coefficient were selected at log (λ) = −2.096.

**FIGURE 4:**
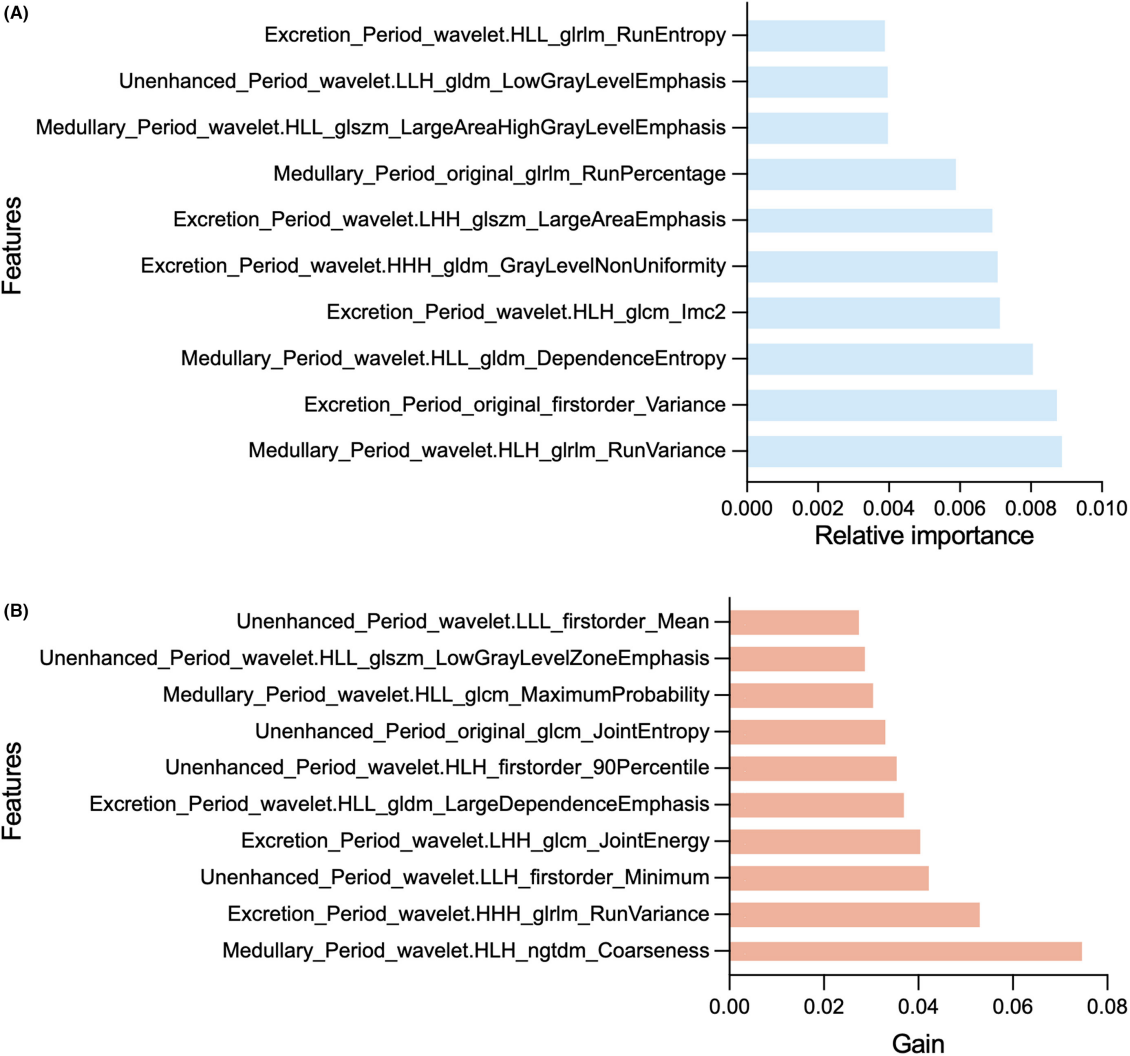
The selection features through random forest (RF) and eXtreme Gradient Boosting (XGboost) machine learning (ML) algorithm. (A, B). The top 10 important features were filtered through RF and XGboost ML algorithms, and the importance of the features gradually increased from top to bottom of the image.

**FIGURE 5:**
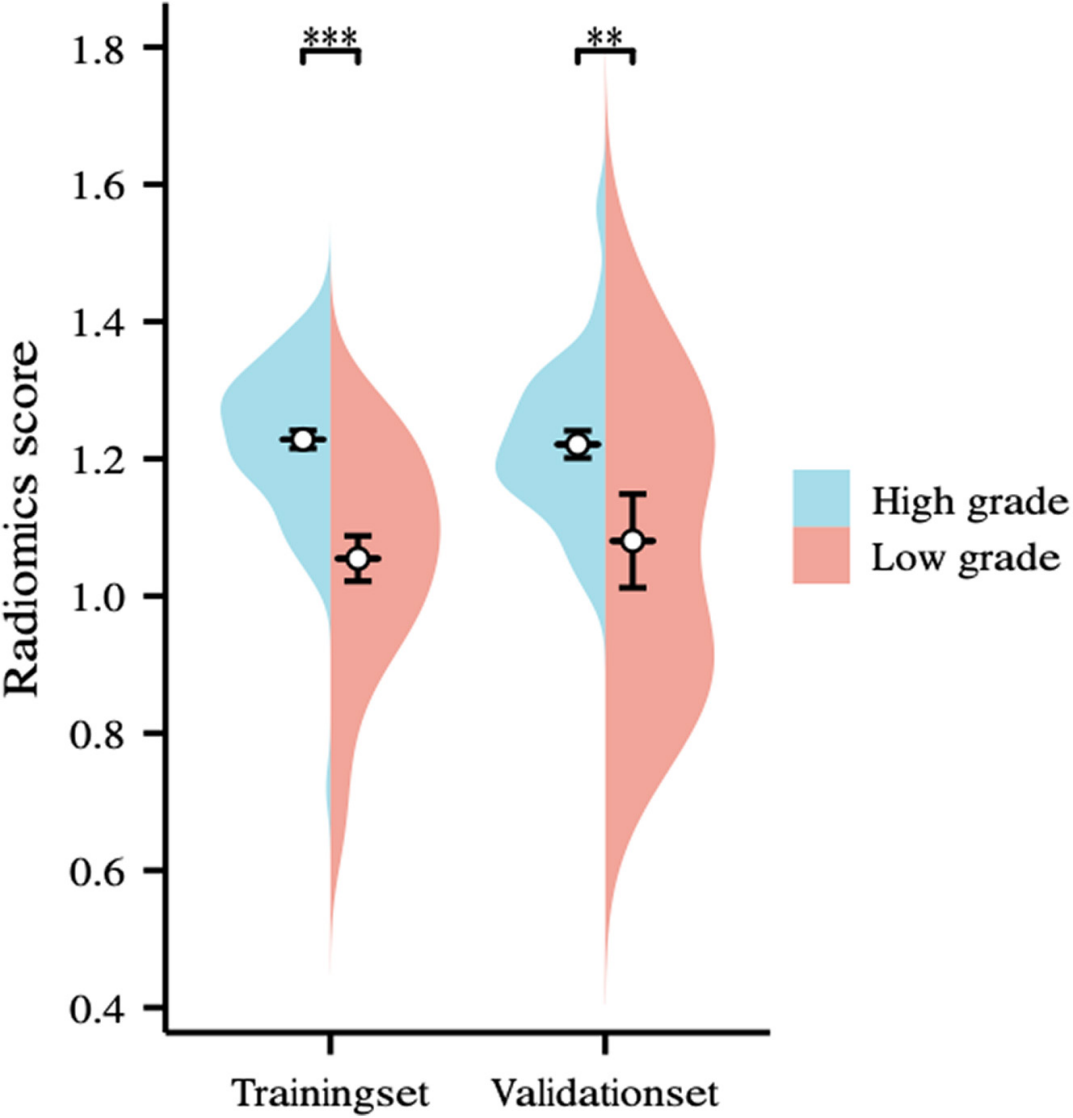
The distribution of radiomics scores based on general‐feature dataset in the training set and validation set. Radiomics scores were significant differences between low‐grade and high‐grade in the training set and validation set, respectively. * *p* < 0.01; ** *p* < 0.001.

The total number of features extracted for each patient included 837 from unenhanced, medullary, and excretion period images, resulting in a cumulative count of 2511 features. By calculating ICC > 0.75, 1079 features were preserved (Appendix S1). After removing redundant features by Pearson and Spearman correlation analysis, 437 features were retained. The remaining 437 features were processed by LASSO algorithm (Figure 3.). Four features were selected and radiomics scores were calculated. In addition, due to the high latitude nature of features, 1079 features were screened again by SVM, RF, and XGBoost ML algorithms. Finally, the three most important features were included (Figure 4., Table 2.). In the clinical baseline characteristics, T‐stage, hydronephrosis, and urine cytology were the independent influencing factors. Hydronephrosis was selected as the best independent influencing factor in this study (Table 3.). In the general‐feature dataset of training set and validation set, there were significant differences in radiomics scores between low‐grade and high‐grade UTUC (both *p* < 0.05) (Figure 5.). Based on the screened features, four sets of feature datasets were created: general‐feature dataset, clinical‐feature dataset, radiomics‐feature dataset, and optimal clinical‐radiomics feature dataset (Table 4.).

**TABLE 2:** Features selection based on four different algorithms.

Models	Features	Coefficient	Relative Importance	Gain
LR	Excretion_Period_wavelet_HHL_firstorder_Mean	0.087	‐	‐
	Medullary_Period_original_glcm_Idmn	0.040	‐	‐
	Unenhanced_Period_wavelet_LHH_glszm_SizeZone NonUniformityNormalized	−0.018	‐	‐
	Unenhanced_Period_wavelet.LHH_glszm_LowGrayLevelZoneEmphasis	−0.067	‐	‐
SVM	Excretion_Period_original_firstorder_Variance	‐	0.237	‐
RF	Medullary_Period_wavelet_HLH_glrlm_RunVariance	‐	0.009	‐
XGBoost	Medullary_Period_wavelet_HLH_ngtdm_Coarseness	‐	‐	0.075

Abbreviations: LR, logistic regression; RF, random forest; SVM, support vector machine; XGBoost, eXtreme Gradient Boosting.

**TABLE 3:** Univariate and multivariate LR analysis algorithm of the clinical baseline characteristics.

Characteristics	Univariate LR analysis			Multivariate LR analysis		
	OR	95%CI	*p*‐value	OR	95% CI	*p*‐value
T‐stage						
Ta	‐	‐	‐	‐	‐	‐
Tis	0.33	0.020–3.030	0.370	0.50	0.020–7.020	0.625
T1	1.45	0.480–4.480	0.508	1.51	0.450–5.270	0.509
T2	19.50	4.540–137.370	<0.001	14.67	3.200–106.980	0.002
T3	7.80	2.400–28.930	0.001	8.81	2.420–37.680	0.002
Hydronephrosis						
No	‐	‐	‐	‐	‐	‐
Yes	7.07	2.610–20.170	<0.001	5.85	1.760–21.420	0.005
Urine cytology						
Negative	‐	‐	‐	‐	‐	‐
Positive	4.29	1.870–10.610	0.001	3.83	1.450–11.050	0.009
Gender						
Femal	‐	‐	‐	‐	‐	‐
Male	1.22	0.530–2.920	0.650	‐	‐	‐
Age	1.00	0.960–1.040	0.992	‐	‐	‐
Tumor location						
Left	‐	‐	‐	‐	‐	‐
Right	1.75	0.790–4.090	0.178	‐	‐	‐
Tumor type						
Renal pelvic	‐	‐	‐	‐	‐	‐
Ureter	1.44	0.660–3.200	0.368	‐	‐	‐

Abbreviations: OR, odds ratio; LR, logistic regression.

**TABLE 4:** The composition of diverse feature datasets.

Dataset type	Series	Construction of dataset
Data 1	General‐feature dataset	Unenhanced_Period_original_shape_SurfaceVolumeRatio, Unenhanced_Period_wavelet_LHH_glszm_SizeZoneNonUniformityNormalized, Medullary_Period_original_glcm_Idmn, Excretion_Period_wavelet_HHL_firstorder_Mean
Data 2	Clinical‐feature dataset	General‐feature dataset, hydronephrosis
Data 3	Clinical‐radiomics feature dataset	Radiomics score calculated based on general‐feature dataset, hydronephrosis
Data 4	Optimal clinical‐radiomics feature dataset	Clinical‐radiomics feature dataset, Excretion_Period_original_firstorder_Variance, Medullary_Period_wavelet_HLH_glrlm_RunVariance, Medullary_Period_wavelet_HLH_ngtdm_Coarseness

### 3.3 Evaluation of four ML models' performance

**FIGURE 6:**
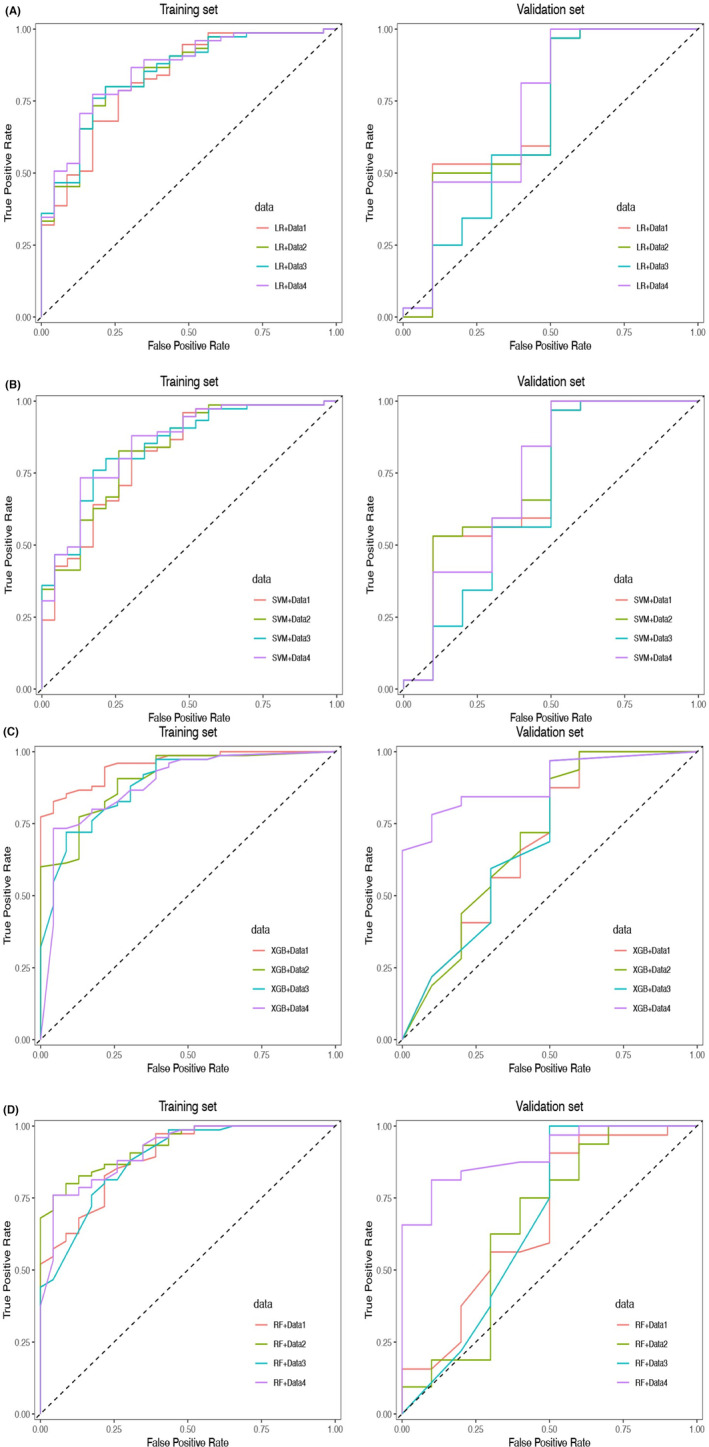
The performance of ML models to predict high‐grade UTUC. (A–D) ROC curves of four kinds of ML models for training set and validation set in different feature datasets (LR, SVM, XGBoost, RF). ML, machine learning; LR, logistic regression; RF, random forest; SVM, support vector machine; XGBoost, eXtreme Gradient Boosting.

The AUC (95% CI), accuracy, sensitivity, and specificity of four ML models across different feature datasets are comprehensively presented in Table 5. ROC for these models in both training and validation sets are illustrated in Figure 6. All 16 ML models demonstrate promising predictive efficacy in predicting high‐grade UTUC. within the validation set. Among them, the RF ML model exhibits superior performance when utilizing optimal clinical‐radiomics feature dataset, achieving remarkable AUC values of 0.914 (95% CI: 0.852–0.977) and 0.903 (95% CI: 0.809–0.997) in the training and validation sets respectively.

**TABLE 5:** Performance of different ML models in diverse feature datasets.

Data	Performances	Models							
		LR		SVM		RF		XGBoost	
		Trainingset	Validationset	Trainingset	Validation set	Trainingset	Validation set	Trainingset	Validationset
Data1	AUC (95% CI)	0.827 (0.729–0.924)	0.716 (0.496–0.935)	0.816 (0.714–0.919)	0.722 (0.507–0.937)	0.893 (0.822–0.964)	0.675 (0.456–0.894)	0.956 (0.919–0.992)	0.691 (0.470–0.911)
	Accuracy	0.847	0.857	0.827	0.857	0.888	0.786	0.878	0.833
	Sensitivity	0.973	0.969	0.987	1.000	0.973	0.875	0.987	0.969
	Specificity	0.435	0.500	0.304	0.400	0.609	0.500	0.522	0.400
Data2	AUC (95% CI)	0.842 (0.751–0.933)	0.706 (0.483–0.929)	0.830 (0.733–0.927)	0.738 (0.526–0.949)	0.930 (0.881–0.979)	0.659 (0.421–0.897)	0.906 (0.842–0.971)	0.706 (0.486–0.927)
	Accuracy	0.837	0.857	0.847	0.881	0.878	0.810	0.878	0.857
	Sensitivity	0.960	0.969	0.987	1.000	1.000	0.938	0.987	1.000
	Specificity	0.435	0.500	0.391	0.500	0.478	0.400	0.522	0.400
Data3	AUC (95% CI)	0.844 (0.755–0.933)	0.672 (0.434–0.910)	0.845 (0.756–0.934)	0.669 (0.429–0.909)	0.886 (0.810–0.962)	0.667 (0.423–0.911)	0.890 (0.817–0.964)	0.694 (0.471–0.916)
	Accuracy	0.847	0.857	0.837	0.833	0.857	0.857	0.847	0.857
	Sensitivity	0.973	0.969	0.973	0.969	0.987	0.969	0.973	0.969
	Specificity	0.435	0.500	0.391	0.400	0.435	0.500	0.435	0.500
Data4	AUC (95%CI)	0.856 (0.770–0.941)	0.725 (0.503–0.947)	0.852 (0.763–0.942)	0.728 (0.505–0.951)	0.914 (0.852–0.977)	0.903 (0.809–0.997)	0.888 (0.808–0.968)	0.892 (0.794–0.990)
	Accuracy	0.837	0.857	0.837	0.857	0.878	0.857	0.867	0.857
	Sensitivity	0.947	0.969	0.973	0.969	1.000	0.969	0.973	0.969
	Specificity	0.478	0.500	0.391	0.500	0.478	0.500	0.522	0.500

Abbreviations: LR, logistic regression; ML, machine learning; RF, random forest; SVM, support vector machine; XGBoost, eXtreme Gradient Boosting.

## 4 DISCUSSION

In this study, we constructed 16 ML models based on the features extracted from the three periods of CTU image and selected the best‐performance ML model to predict high‐grade UTUC. In addition, we compared the performance of the four kinds of ML models by constructing different feature datasets. The results showed that all the ML models had good classification performance, and the ML model based on RF algorithm had the best prediction performance, with the highest AUC values in the training set and validation set respectively 0.914 (95% CI: 0.852–0.977) and 0.903 (95% CI: 0.809–0.997). This model can be used as a non‐invasive method for predicting preoperative pathological grade of UTUC.

Currently, alongside invasive ureteroscopy and biopsy, radiological imaging plays an indispensable role in the diagnosis, staging, and follow‐up of suspected upper urinary tract lesions.^20^ Among various radiological examination methods, CTU is the preferred imaging modality.^21^ Despite its high diagnostic sensitivity and specificity in identifying space‐occupying lesions of the upper urinary tract, it still lacks accuracy in tumor grading. Therefore, we aimed to develop and validate a non‐invasive ML model capable of accurately determining the pathological grade of UTUC. In 2012, Lambin et al. introduced the concept of radiomics, which obtains digital information inside tumors through images.^22^ With the advancement of an increasing number of ML algorithms, radiomics research has gained widespread utilization in disease diagnosis and patient prognosis assessment.^23^ In a study similar to ours, the AUC value of the validation set was 0.860 (95% CI: 0.742–0.979) and the diagnostic accuracy was 0.838 for the prediction of pathological grade of bladder cancer based on CT radiomics.^24^ Mammen et al. demonstrated significant differences in tumor texture between low‐grade and high‐grade UCC (*p* = 0.03) by applying CT texture analysis (CTTA) to distinguish the pathological grade of UTUC.^25^ These studies have established the feasibility of CT‐based radiomics for pathological grades. However, the extracted texture features were derived from both unenhanced and enhanced CT images, without fully exploiting the available information in CT imaging data. Furthermore, an increasing number of studies are employing diverse ML algorithms for feature selection and model construction.^26^, ^27^ Unfortunately, there is a paucity of studies investigating the application of this method for predicting the pathological grade of UTUC. In this study, we have developed a model that exhibits robust predictive ability. Additionally, we have explored various algorithms to construct feature datasets and identify the most representative radiomics datasets.

Due to the limited resolution of CT scans for soft tissue, it poses a challenge to visually distinguish tumor tissue components on CT images in clinical practice. Consequently, there is a scarcity of radiomics assessments utilizing CT for pathological tissue grading in UTUC. Conversely, owing to the superior high‐resolution capabilities of MRI in detecting soft tissue abnormalities, MRI sequences exhibit commendable diagnostic performance when identifying pathological grade.^28^ However, MRI examination is associated with prolonged scanning time, contraindications for certain patients, and relatively high costs. Consequently, the utilization of MRI for evaluating UTUC may not be suitable for all individuals. In cases where patients present with initial symptoms of low back pain or hematuria without obvious causative factors, CTU examination is more commonly employed compared to MRI. A radiomics ML model based on multidetector CT (MDCT), developed by Moldovanu et al., demonstrated excellent performance in predicting the pathological grade of renal tumors, with an AUC of 0.99 (95% CI: 0.92–1.00) in the validation set.^29^ This study highlights the significant potential of radiomics features derived from multimodal CT imaging for the accurate prediction of tumor pathology.

In our study, among the four ML models constructed using Data1, the SVM model exhibits superior predictive performance in the validation set. Several research that used CTTA to create a variety of ML models for tumor pathology grade prediction have consistently shown that SVM performs better than other ML models, which is in line with our findings.^30^, ^31^ Furthermore, the inclusion of clinical baseline characteristics significantly enhanced the model's predicted performance. In line with our findings, a study demonstrated a significant association between hydronephrosis and the diagnosis of ureteral tumors (*p* = 0.0307).^32^ Notably, our study encompassed a larger sample size, enhancing our results' reliability.

The method of computational radiomics scores performs well in most cases for constructing nomograms for disease diagnosis or evaluating prognostic risk.^33^, ^34^, ^35^ Our results show that taking into account the clinical‐radiomics feature dataset (Data 3) does not significantly improve the contribution of radiomics scores to model performance compared to the uncomputed clinical‐feature dataset (Data 2). In contrast to our findings, a prior study has demonstrated the potential of LG ML models based on MDCT computed radiomics scores in enhancing predictive performance for renal tumor pathological grade.^29^ However, our LR ML model did not yield similar outcomes. This discrepancy may be attributed to research subjects, divergent approaches to segment ROI, and distinct characteristics of the model.

**FIGURE 7:**
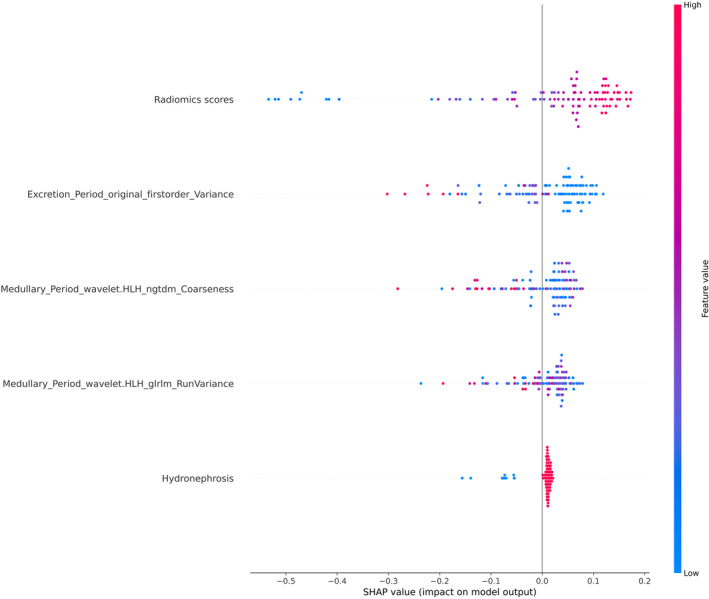
Figure of Shap value. Rank the importance of each feature in the output of the random forest (RF) machine learning(ML) model. Different colors (red and blue) indicate different degrees of influence on the model output.

To investigate the feature importance order of the optimal clinical‐radiomics dataset in the RF model, we computed SHAP values to determine the ranking of features (Figure 7).^36^ Feature of “mean” derived from the first order primarily represents the average grayscale intensity within the ROI. In the excretion period of CT image, the average grayscale intensity is generally higher compared to that in the non‐enhanced period due to tissue absorption of contrast agents.^11^ Zhang et al. employed CTTA to discriminate between low‐grade and high‐grade urothelial carcinomas. The study revealed significant differences in the feature of “mean” on unenhanced and contrast‐enhanced CT images for low‐grade urothelial carcinomas (*p* < 0.001), with unenhanced images exhibiting a lower feature of “mean”.^37^ The dose of contrast agent may have an effect on the gray value, however, this effect is not yet clear and more research is needed. The texture feature of GlCM assesses the textural characteristics of an image by analyzing the spatial alignment statistics of pixel intensity.^38^ Numerous studies have substantiated the significance of GLCM features in assessing tumor heterogeneity. For instance, GLCM correlation has been employed to predict renal cell carcinoma with tumor thrombus,^39^ while GLCM “clustershade” has been utilized for prognosticating pancreatic neuroendocrine tumor pathological grade and gastrointestinal stromal tumor prognosis.^40^, ^41^ “Inverse difference moment normalized” (idmn) is used to evaluate the local homogeneity of the image. In this study, we observed a potential association between GLCM idmn and the pathological grade of UTUC. In patients with breast cancer, a substantial association has been shown between GLCM idmn and one‐year relapse‐free survival.^42^ Moreover, it was discovered that GLRLM may be used to predict acute pulmonary thromboembolism,^43^ whereas NGTDM is linked to the prediction of pathological grade in bladder cancer.^44^ The aforementioned studies have demonstrated that radiomics features not only enable the expression of tumor heterogeneity, which is challenging to detect visually, through digital information but also exhibit potential for evaluating tumor prognosis.^12^ In radiomics research, acquisition of high‐quality and artifact‐free radiographic images is a prerequisite, while variations in image acquisition mode, matrix size, and algorithm construction may impact the accuracy or generalizability of radiomics features or models.^45^ With the continuous advancement of research, an increasing number of representative features are gradually demonstrating a positive correlation with tumor heterogeneity, thereby highlighting the potential for detecting highly correlated features as a predictive method for tumor pathological grading.

There are several limitations in our study. First, due to the retrospective design, potential selection bias may exist; therefore, further studies with a larger sample size and more comprehensive research are warranted. Second, this study's modeling data utilized originated solely from a single center investigation and has not undergone external validation. To ascertain the model's generalizability, external validation must be done gradually. Third, the sample size of the study is relatively small, necessitating additional data samples to enhance classification performance of the model. Lastly, potential influences on results could arise from factors such as image acquisition mode, reconstruction parameters, tumor segmentation method, feature selection, and so on.

In conclusion, the CTU‐based ML model holds great promise in predicting the pathological grade of UTUC. As a preoperative evaluation tool for UTUC's pathological grade, the radiomics model can serve as an invaluable supplementary method to ureteroscopic biopsy.

## AUTHOR CONTRIBUTIONS

**YangHuang Zheng:** Conceptualization (equal); data curation (equal); formal analysis (lead); software (equal); writing – original draft (lead). **Hongjin Shi:** Conceptualization (equal); data curation (equal); methodology (equal); software (equal); visualization (equal). **Shi Fu:** Conceptualization (equal); data curation (equal); methodology (equal); supervision (equal). **Haifeng Wang:** Conceptualization (equal); data curation (equal); methodology (equal); supervision (equal). **Jincheng Wang:** Conceptualization (equal); methodology (equal); software (equal); visualization (equal). **Xin Li:** Data curation (equal); methodology (equal); supervision (equal). **Zhi Li:** Data curation (equal); methodology (equal); supervision (equal). **Bing Hai:** Conceptualization (equal); data curation (equal); methodology (equal); supervision (equal); writing – review and editing (equal). **Jinsong Zhang:** Conceptualization (equal); data curation (equal); methodology (equal); supervision (equal); writing – review and editing (equal).

## FUNDING INFORMATION

Not applicable.

## CONFLICT OF INTEREST STATEMENT

The authors declare no competing interests.

## ETHICS STATEMENT

The study was conducted according to the guidelines of the Declaration of Helsinki and approved by the Institutional Review Board of the Second Affiliated Hospital of Kunming Medical University, which exempted from ethical review and patient informed consent.

## INFORMED CONSENT STATEMENT

The Institutional Review Board of the Second Affiliated Hospital of Kunming Medical University has approved this study and waived the requirement for patient informed consent.

## Supporting information


Appendix S1.

## Data Availability

The data that support the findings of this study are available from the corresponding author upon reasonable request.
