# Supplementary material for: A computed tomography urography‐based machine learning model for predicting preoperative pathological grade of upper urinary tract urothelial carcinoma
Source: Cancer Med. 2024 Jan 4;13(1):e6901. doi: 10.1002/cam4.6901 (PMC10807597; doi:10.1002/cam4.6901)
Supplement: Supplementary file 1 — Appendix S1. [file CAM4-13-e6901-s001.docx]

**CTU examination.** (Appendix A .)

The patients were subjected to a fasting period of 4-6 hours before the CTU examination, during which they refrained from eating or drinking. Additionally, they provided informed consent for the administration of contrast agents. To ensure proper bladder filling, they drank 1000ml of water within 1-2 hours before the examination. The imaging was performed using CT systems from TOSHIBA, PHILIPS, and SIEMENS scanning machines. The imaging parameters included a tube voltage of 120kV, tube current of 350mA, layer spacing and thickness of 1mm each, and a matrix size of 512*512 pixels. Following an initial unenhanced scan, a nonionic contrast agent (iohexol - Onpac China) was injected through the elbow venous group at a flow rate of 3-4ml/s using a double-barrel high-pressure injector. The dose administered ranged between 80-120ml. Before scanning, the tube was flushed with saline solution (18ml). A delay in scanning by approximately 25-30 seconds occurred for cortical period imaging after injecting the contrast agent; for medullary period imaging it was delayed by around 55-60 seconds; and for excretory period imaging it was delayed by about 7.5-10 minutes. The obtained CTU image data were reconstructed with a layer thickness and interval set at 1.0 mm each and exported in DICOM format.

**Procedure and results of urological cytology.** (Appendix B.)

1. Collect more than 50ml of fresh midstream urine and transfer it into a centrifuge tube. Centrifuge the sample at 2000 revolutions per minute for 5 minutes.
2. To isolate cells from the urine, discard the supernatant and perform a washing step by adding 30 ml of cleaning solution to the sediment. Place the mixture on a washing oscillator set at 2000 revolutions per minute for 5 minutes.
3. Transfer the liquid obtained after oscillation into another centrifuge tube. Centrifuge this sample at 2000 revolutions per minute for 10 minutes, remove the supernatant, smear the sediment onto slides, and allow them to air dry at room temperature.
4. After drying, fixate with anhydrous alcohol for 15-20 minutes followed by rinsing with distilled water two or three times.
5. Once fixed, stain specimens in a hematoxylin staining solution for five minutes.
6. After the staining is completed, use the flowing water to wash the hematoxylin for 5 to 10 seconds.
7. Continue to use 0.5% hydrochloric acid alcohol to clean the slide for 10 seconds.
8. Wash the slide with distilled water for 5 seconds and then immerse it in dilute lithium carbonate solution for 1-2 minutes, and place it at room temperature.
9. Use 0.125% eosin solution for deep staining, which needs to be completely covered on the slide, and process it at room temperature for 1 minute.
10. After the slide is encapsulated, use a microscope to observe.

**Diagnostic Categories for The Paris System for Reporting Urinary Cytology**

1. Nondiagnostic/unsatisfactory;
2. Negative for high-grade urothelial carcinoma (NHGUC);
3. Atypical urothelial cells (AUC);
4. Suspicious for high-grade urothelial carcinoma (SHGUC);
5. High-grade urothelial carcinoma (HGUC);
6. Low-grade urothelial neoplasm (LGUN);

Other: primary and secondary malignancies and miscellaneous lesions.

**Figure of ICC.** (Appendix C.)
